# Prevention Better Than Cure

**Published:** 1913-02

**Authors:** C. D. Rice

**Affiliations:** Austin, Texas


### Prevention Better Than Cure.

BY MRS. C. D. RICE, AUSTIN, TEXAS.

The tendency of the day is to prevent disease rather than seek a cure after it has been contracted. For this reason we should gladly pay a physician who is willing to give us the advantage of his superior knowledge of the laws of health to protect us from sickness and suffering. This would be far better than to wait until disease has fastened its hold upon us, necessitating the continued service of the physician, the endurance of much pain, suffering, loss of time and general unfitness for life's duties. Through medical journals, lectures, and the press generally, the public is being informed as to methods of preventing disease. Our eyes are being opened, so to speak, and there is no way to estimate the good that is beig done in this direction. We know now that the public drinking cup is to be avoided and that mosquitoes and flies are our enemies. We will not mention here the many other danger signals that the medical profession has given the public to aid in the preservation of the good health which we all so much desire and have a right to possess.

This article is written for the sake of emphasizing one special danger which the writer thinks is important, but which, heretofore, has been carelessly overlooked. As soon as it is mentioned, all thinking people will, no doubt, agree that a movement to abolish its use is absolutely necessary to public health. I speak of the pocket handkerchief. Oh, those germ-laden pockets and pocket handkerchiefs! Nine times out of ten every bad cold can be traced to the unsanitary handkerchief to which you have been exposed. It may have been in some public gathering, on a street car, train, or while entertaining a friend, where you were forced to sit by or be near a person who, having a cold, used a handkerchief continually. Could you have thrown a ray of bright light upon the small dust particles that came from the dried secretions on the handkerchief as it was shaken and handled, you would have seen millions of little particles distributing themselves in the air you were breathing. Those dust particles, breathed through your nasal passage, found a lodging place that furnished a fertile soil for the cold germs. Perhaps they did not take root at that time, but you were, nevertheless, exposed. If, for some reason, you had been extremely tired or not in good condition, you would certainly have had to suffer the consequences. We all know that a cold is not a simple matter. It often leads to serious troubles. This manner of catching cold does not seem to be generally recognized, but the writer has had the experience sufficiently often to believe in it, and hence feels it a sense of duty to warn others.

In the methods of preventing tuberculosis, we are told to beware of the feather duster and not to sweep, where the disease is found, among careless people, without using certain precautions, since it is well known that the tuberculosis germ found in the dried sputum, is, by dusting and sweeping, thrown into the air and with the air, breathed into the lungs. Let us add to this precaution the importance of protecting ourselves from the danger of dust from a handkerchief which has upon it the dried secretions from the nose, throat or mouth of a consumptive. We should not forget that one such handkerchief, when drawn from the pocket, handled or moved about, scatters the dust which causes the real trouble when breathed into the lungs of a susceptible person.

We might go further and mention other horrible diseases, in the distribution of which this one little pocket handkerchief plays a very important part. The danger in the use of the public drinking cup furnishes no greater cause for alarm. Catarrh, sore throat, la grippe and other troubles might be greatly lessened if there were no such thing as a pocket handkerchief.

The so-called "Chautauqua salute," in which each member of a large audience waves a handkerchief in salute to some popular speaker, is a beautiful and inspiring sight, but nevertheless a dangerous practice. Did you ever listen to the general commotion just after one of those demonstrations—such coughing and sneezing and clearing of throats! Oh, what troubles were in the air! In a large gathering, where thousands of people have assembled, the germ-laden handkerchiefs are ever present, and to frantically wave them in the air is, to say the least, a dangerous method of distributing germs.

When we find that we have contracted a cold, let us stay away from public gatherings and in every possible manner avoid the use of handkerchiefs and substitute for them a soft paper or something that can be burned or carried away by sewerage. Let us remember that the handkerchief helps to spread many diseases and that "the occurrence of any disease which can be prevented is some one's fault."

Can we not all join our forces and never cease our efforts until we have found ways and means to abolish the use of the pocket handkerchief?

